# A compact variable stiffness joint for compliant robotics enabled by torsion-spring mean-diameter variation

**DOI:** 10.1038/s41598-026-50967-4

**Published:** 2026-05-05

**Authors:** Xiangxu Qu, Zhengkai Feng, Kang Ju, Cui Liu, Lan Chen, Ruiqi Xu, Yuanbo Yang, Gang Wang, Yuting Zhang, Jingjie Dai

**Affiliations:** 1 College of Mechanical and Electrical Engineering, Qingdao Binhai University, Qingdao, 266555 China; 2 Dalian Institute of Industrial Software, Dalian, 116014 China; 3 School of Mechanical Engineering, Hebei University of Technology, Tianjin, 300131 China

**Keywords:** Variable stiffness joint, Torsion spring, Compliant actuation, Human-robot interaction, Engineering, Physics

## Abstract

For robotic applications requiring compliance and safety in rehabilitation training, physical human–robot collaboration, and unstructured environments, this study proposes a variable-stiffness joint design method, termed TSDV, based on the mean-coil-diameter variation mechanism of a torsion spring. First, the variation of the mean coil diameter of a cylindrical torsion spring with joint deflection angle is analyzed. On this basis, a structural scheme for stiffness modulation is proposed by constraining the inward contraction of the spring inner diameter. The joint mainly comprises a torsion spring, an internal slotted sleeve, and a ball-guiding sleeve, and features a compact architecture, a wide stiffness regulation range, coaxial alignment with the robot joint axis, and both passive and active stiffness modulation modes. Subsequently, a nonlinear stiffness model of the variable-stiffness joint system is established using an energy-based method, and the validity of the theoretical model is verified through numerical simulations and finite-element analysis. The results show that both the output torque and the joint stiffness exhibit tunable nonlinear characteristics with respect to the deflection angle and regulation parameters. Finally, experiments are conducted on a TSDV prototype. The experimental results demonstrate that, under motor actuation, the proposed joint can achieve both passive and active stiffness regulation. When the deflection angle reaches 1.2 rad, the output torque range increases from 0 to 3.8 Nm to 0–9.2 Nm. Moreover, when the slotted sleeve fully suppresses the variation of the torsion spring inner diameter, the joint exhibits a locking function and transitions into a rigid joint. These findings indicate that the proposed variable-stiffness joint offers a compact structure, a large stiffness regulation range, and high control resolution, thereby providing a new approach for safe interaction and compliant actuation in robotic systems.

## Introduction

Traditional industrial robots are typically developed with high structural stiffness and high-precision position control, enabling fast, accurate, and repeatable trajectory tracking. However, as robotic applications continue to expand into unstructured environments such as human–robot interaction, rehabilitation training, and emergency rescue, safety and adaptability have gradually become primary design objectives of actuation systems^1–3^. To reduce collision impacts, improve interaction compliance, and enhance adaptability to uncertain external loads, elastic elements are often introduced into the drive chain, thereby forming compliant actuators with controllable compliance characteristics. Among them, variable stiffness joints can adjust the equivalent joint stiffness according to task requirements and operating conditions, and have therefore been increasingly applied in robotic systems operating in unstructured environments^4^.

From the perspective of structural arrangement, variable stiffness joints can generally be classified into two categories: serial and antagonistic. In serial configurations, elastic elements are typically placed between the motor output and the load, and the equivalent stiffness is regulated by altering structural geometry or the force transmission ratio. In antagonistic configurations, elastic elements are arranged in parallel, and the equivalent stiffness is adjusted through the combined action of the elastic elements on both sides. The operating principles of serial variable stiffness joints mainly include preload adjustment, transmission-ratio adjustment (e.g., cam- or lever-based mechanisms), modification of the equivalent characteristics of elastic elements (e.g., effective length variation, flexure hinges, and configuration reconfiguration), and electromagnetic approaches. In contrast, antagonistic schemes mainly include cable-driven, pneumatic artificial muscle, and multi-elastic-element coordinated actuation forms^5–8^.

Among serial variable stiffness joints, cam-based solutions have been widely adopted because of their compact structures and controllable force transmission relationships. Representative designs include the jumping-robot joint L-MESTRAN, which achieves stiffness variation by adjusting the roller–cam contact slop^9^, and the PLVL-VSA, which realizes variable stiffness through the coupled adjustment of the rope–cam mechanism and the moving lever arm point^10^. Furthermore, reconfigurable cam-slot and cam-profile designs enable simultaneous optimization of transmission ratio, load distribution, and stiffness regulation performance at the mechanism level, thereby improving stiffness adjustment efficiency and regulation range^11^. In addition, comparative analyses and prototype tests have been carried out on key aspects such as cam profile design, harmonic drive matching, and roller contact characteristics, further enhancing the reliability and engineering applicability of such joints^12,13^.

Lever-based solutions usually realize continuous stiffness regulation by moving the spring action point or the pivot position, thereby changing the moment arm of the elastic element with respect to the output end and the equivalent transmission ratio. AwAS can regulate stiffness at the equilibrium position without changing spring preload, and the adjustment displacement is approximately perpendicular to the spring force direction, thus reducing the energy consumption of stiffness regulation^14^. AwAS-II and MOD-AwAS further optimize the transmission structure and damping characteristics, making the stiffness adjustment process smoother and easier to control^15,16^. Onda et al. adopted a lever–wire transmission mechanism to regulate the stiffness and posture-holding capability of a compliant structure^17^. Wang et al. employed a planetary gear–lever coupling mechanism and helical spring preload to achieve stiffness regulation toward predefined targets^18^. Shao et al. realized large-range continuous stiffness adjustment by moving the pivot to alter the equivalent lever arm^19^. Milazzo et al. adjusted tendon tension through a lever arm to regulate the stiffness of a three-degree-of-freedom wrist joint, and also established the corresponding modeling and control methods^20^.

On the other hand, variable stiffness can also be achieved by modifying the equivalent characteristics of elastic elements. Leaf-spring or flat-spring-based schemes enable continuously adjustable joint stiffness through the design of structural parameters and deformation modes under loading^21–23^. Flexure-hinge-based solutions can achieve a relatively wide stiffness variation range within a small structural size, but they are more prone to hysteresis and nonlinear errors during loading and unloading processes^24^. In addition, the RVSA employs torsional elastic elements and S-shaped spring sets to realize continuous regulation by varying the effective working length^25^, while the JASR adopts switchable mechanism configurations to accommodate different stiffness and motion requirements under different operating conditions^26^.

In studies on antagonistic variable stiffness joints, the core issue mainly concerns the relationship between stiffness regulation and posture adjustment under antagonistic layouts^27^. Early work established the basic framework for coordinated regulation of joint stiffness and position through antagonistic actuation^28,29^. Subsequently, bidirectional antagonistic variable stiffness actuators were further improved in terms of structural design and implementation details, and gradually evolved into a general scheme suitable for integration into multi-degree-of-freedom systems^30^. Meanwhile, considering that antagonistic structures simultaneously affect torque output and the adjustable stiffness range, relevant studies have improved the operating range and performance distribution of both through nonlinear elastic design and transmission-layout optimization^31,32^. In terms of actuation form, solutions such as extensor–contractor pneumatic artificial muscles (ECPAMs) achieve variable stiffness by combining pneumatic actuation with antagonistic layouts, but they also introduce increased system complexity due to air supply and pressure regulation requirements^33^. In the field of soft actuators, approaches have also emerged that alter the equivalent material and structural stiffness through pneumatic or fluidic structures, including pneumatic actuators whose stiffness varies with pressure through wedge-shaped structures^34,35^. In addition, designs that couple the variable-radius principle with antagonistic mechanisms, such as the VSA-AHLM, have shown that a large stiffness range can be achieved in a compact structure by coordinated adjustment of geometric radius and preload^36^. Systems such as ASRA, which use multiple pairs of linearly antagonistic springs, place greater emphasis on structural simplicity and controllability of stiffness regulation^37^.

A comparison of different types of variable stiffness joints shows that antagonistic variable stiffness joints generally have larger structural dimensions, but under specific configurations their output torque can be superposed as the sum of the torques of two motors, thus providing higher load-carrying capability. In contrast, serial joints exhibit more prominent performance in key aspects such as torque output and stiffness regulation, while also featuring more compact structures and easier integration, and are therefore more widely used. Among them, cam-based variable stiffness joints are compact and have small gaps in contact-based force transmission, but they impose higher requirements on manufacturing and assembly accuracy and usually exhibit strong stiffness nonlinearity. Lever-based joints can more readily realize a prescribed torque–angle relationship, but their overall size is relatively large, and their stiffness adjustment range is often constrained by geometric stroke limitations.

Although various implementations of variable stiffness joints have been proposed through preload adjustment, transmission-ratio regulation, and elastic-element characteristic modulation, most existing studies focus primarily on adjustment mechanisms and transmission relationships, and often treat elastic elements as energy-storage components with fixed parameters^38–40^. In fact, under the demand for maintaining structural compactness while enlarging the variable stiffness regulation range, exploring stiffness variation mechanisms from the intrinsic physical-property changes of the elastic elements themselves offers a promising direction. In particular, when torsional elastic elements operate under large angular deflections, their geometric parameters and equivalent mechanical properties vary with deformation, which makes it possible to introduce new variable stiffness mechanisms without significantly increasing the complexity of the transmission chain. Based on the physical characteristic that the coil diameter of a cylindrical torsion spring changes during twisting, this paper proposes a compact variable stiffness joint mechanism, termed TSDV, featuring a simple transmission path and suitability for large angular deflections. The proposed mechanism possesses both passive and active variable stiffness capabilities, which can significantly improve the resolution and controllability of stiffness regulation. Meanwhile, its rotational axis is aligned with the robot joint axis, allowing direct series integration with robotic joints and providing fundamental support for safe interaction in collaborative tasks involving direct human contact as well as in complex unstructured environments.

The main contributions of this study are as follows. First, by exploiting the physical characteristic that the mean coil diameter of a torsion spring varies during twisting, a novel variable-stiffness joint is proposed in which stiffness modulation is achieved by constraining the contraction of the spring inner diameter. Second, a nonlinear joint torque model incorporating radial constraint is established, thereby revealing the mechanism by which radial supporting force influences the output torque and stiffness characteristics of the joint. Third, the validity of the theoretical model is verified through finite-element analysis and prototype experiments, and the stiffness variation of the joint under different regulation parameters is systematically analyzed.

## Methods

**Fig. 1:**
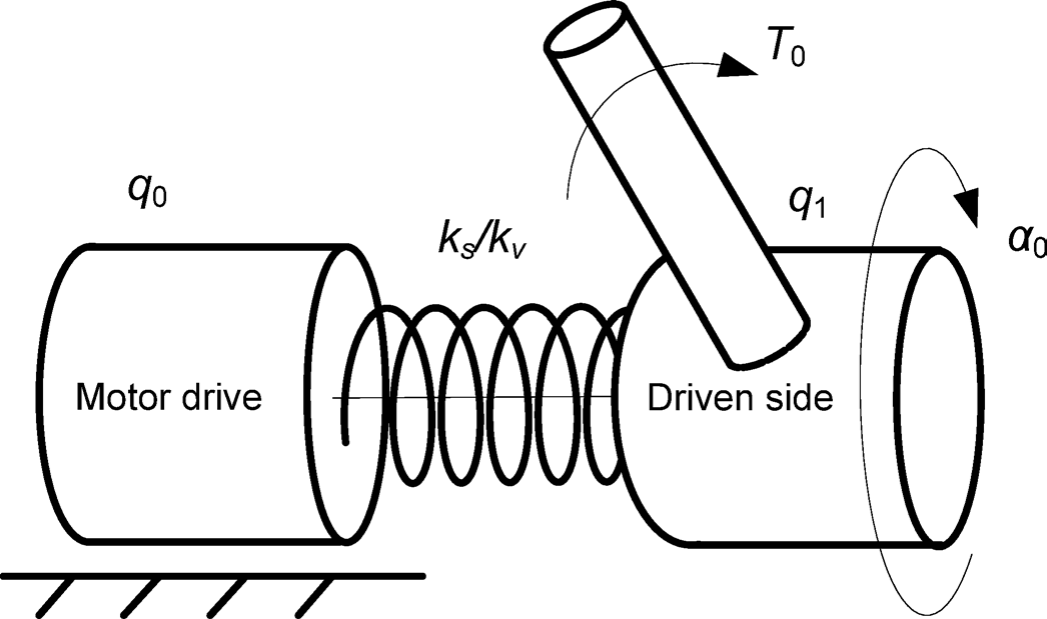
Schematic illustration of a flexible actuator.

Compliant joints, including both constant-stiffness and variable-stiffness types, can be abstracted as a transmission port connecting the driving side $\:{q}_{0}\:$and the actuator side $\:{q}_{1}$, as shown in Fig. 1. According to whether the equivalent stiffness is adjustable, they can be classified into two categories:

(1) Constant-stiffness compliant joints (series elastic actuators), in which a flexible element with fixed stiffness is connected in series between $\:{q}_{0}\:$and $\:{q}_{1}$, and the equivalent stiffness $\:{k}_{s}\:$remains approximately constant. Their main advantages lie in passive compliance and impact resistance. However, their limitation is that the stiffness cannot be adjusted, making it difficult to simultaneously achieve both compliance and control bandwidth.

(2) Variable-stiffness compliant joints, in which the equivalent stiffness $\:{k}_{v}\:$is regulated through a mechanical mechanism and decoupled from position control. The maximum output torque of the joint is determined by the mechanical properties of the compliant element and the output capability of the driving motor. Because $\:{k}_{v}$is adjustable, the joint exhibits a nonlinear torque–angle characteristic.

Methods for achieving the desired stiffness variation through the design of nonlinear elastic elements are generally complex, and the stiffness accuracy strongly depends on the performance of the actuation mechanism and the selected materials, resulting in relatively high cost. In contrast, variable stiffness actuator design approaches based on linear elastic elements allow the desired nonlinear output characteristics to be realized more accurately, while the required linear elastic components are more readily available. Therefore, this study adopts a design scheme in which nonlinear torque characteristics are generated on the basis of a constant-stiffness torsion spring.

### Variation law of the mean coil diameter of the torsion spring

Cylindrical helical torsion springs are commonly used in applications such as force measurement, buffering, and energy storage. In this section, another loading and deformation mode is considered. When both ends of the torsion spring are twisted, the spring rotates correspondingly about its axis, and each coil gradually wraps more tightly around the inner cylindrical shaft, thereby effectively increasing the spring stiffness. It is assumed that the wire diameter of the torsion spring remains unchanged, while the developed length along its centerline is approximately constant^41^. Let the initial number of active coils, initial mean coil diameter, and angular deflection of the torsion spring be denoted by $\:{n}_{1}$, $\:{D}_{1}$, and $\:\theta\:$, respectively. Then, the developed length $\:L\:$of the spring can be expressed as:

1 $$L={n_1}{\pi}{D_1}$$

The number of active coils after twisting becomes:

2 $${n_2}=\frac{{2\pi{n_1}+\theta }}{{2{\text{}}\pi}}$$

The mean coil diameter of the torsion spring after twisting is:

3 $$ {D_2}=\frac{{{n_1}{D_1}}}{{{n_2}}}$$

Since the developed length $\:L\:$remains unchanged, the variation in the mean coil diameter of the torsion spring can be expressed as:

4 $$\Delta D(\theta )={D_1} - {D_2}=\frac{{\theta {D_1}}}{{2{\pi}{n_1}+\theta }}$$

When the reduction in the inner radius is equal to the initial radial clearance $\:{\delta\:}_{0}$ between the inner coil of the torsion spring and the sleeve during assembly, the torsion spring just comes into contact with the slotted sleeve, and the corresponding torsion angle $\:{\theta\:}_{c}$can be expressed as:

5 $${\theta _c}=\frac{{4{\pi}{n_1}{\delta _0}}}{{{D_1} - 2{\delta _0}}}$$

**Fig. 2:**
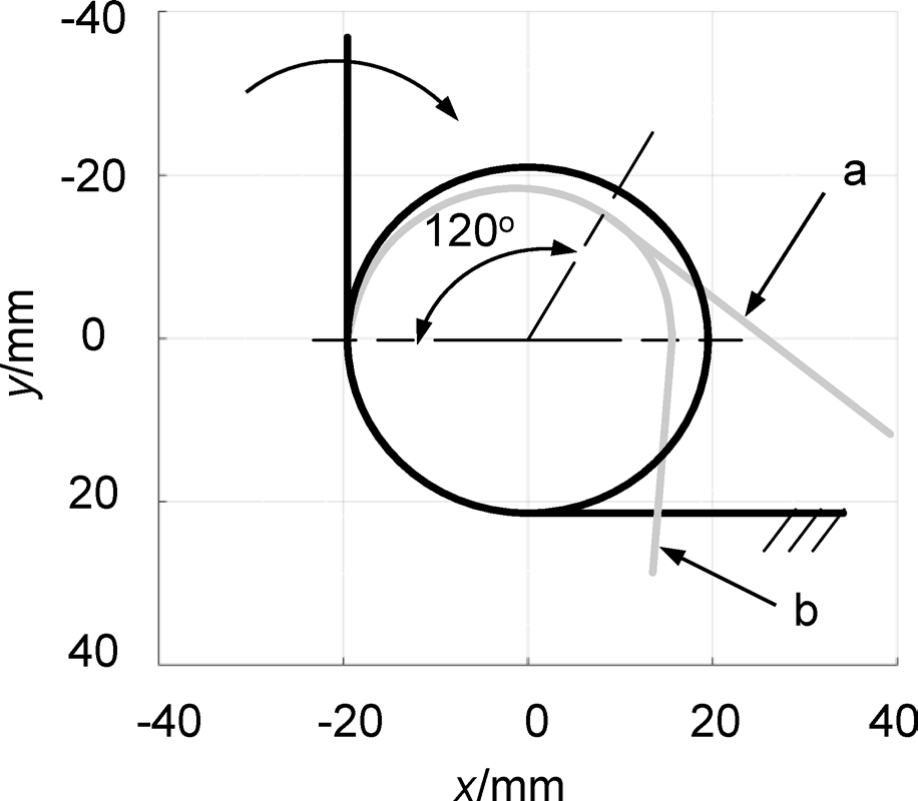
Schematic of the variation in the mean coil diameter of the torsion spring.

As indicated by Eq. (4), the mean coil diameter of the torsion spring is determined by the number of active coils and the angular deflection of the spring under external loading. To evaluate this relationship, a torsion spring with $\:{D}_{1}=40{\hspace{0.17em}mm}\text{}$and $\:{n}_{1}=10\:$is taken as an example, and the variation in the mean coil diameter is shown in Fig. 2.

In Fig. 2, one end of the torsion spring is fixed, and the $\:x$- and $\:y$-coordinates represent the horizontal and vertical distances from the spring center, respectively. The thick black solid curve denotes the initial position of the torsion spring during deflection, while the thin gray solid curve represents the actual position of the free end of the spring. Point $\:a$indicates the position of the free end when the deflection angle reaches $\:{120}^{\circ\:}$, at which the mean coil diameter decreases by approximately 2.67 mm, from 40 mm to about 37.3 mm. Point $\:b$denotes the position of the free end when the deflection angle reaches $\:{180}^{\circ\:}$, where the mean coil diameter decreases by approximately 4.0 mm, from 40 mm to about 36.0 mm. As shown in the figure, the trajectory of the free end of the torsion spring is not a standard circular arc, but rather an elliptic-like arc path that gradually contracts and moves toward the center.

### Conceptual design

According to the variation law of the mean coil diameter of the torsion spring described in Eq. (4), this paper proposes a new approach for realizing variable stiffness, namely, enhancing the joint output torque by restraining the reduction in the mean coil diameter of the torsion spring. The joint employs a cylindrical torsion spring as its core elastic element, with the input and output ends rigidly connected to the two sides of the robot revolute joint, respectively. When relative deflection occurs between the drive side and the output side, the torsion spring undergoes torsional deformation, thereby producing compliant output at the joint while storing passive elastic potential energy in the elastic element. As a result, the joint can not only absorb impact and improve operational safety under contact and collision conditions, but also release the stored elastic potential energy during periodic motion, thereby enabling a certain degree of energy recovery and energy saving.

**Fig. 3:**
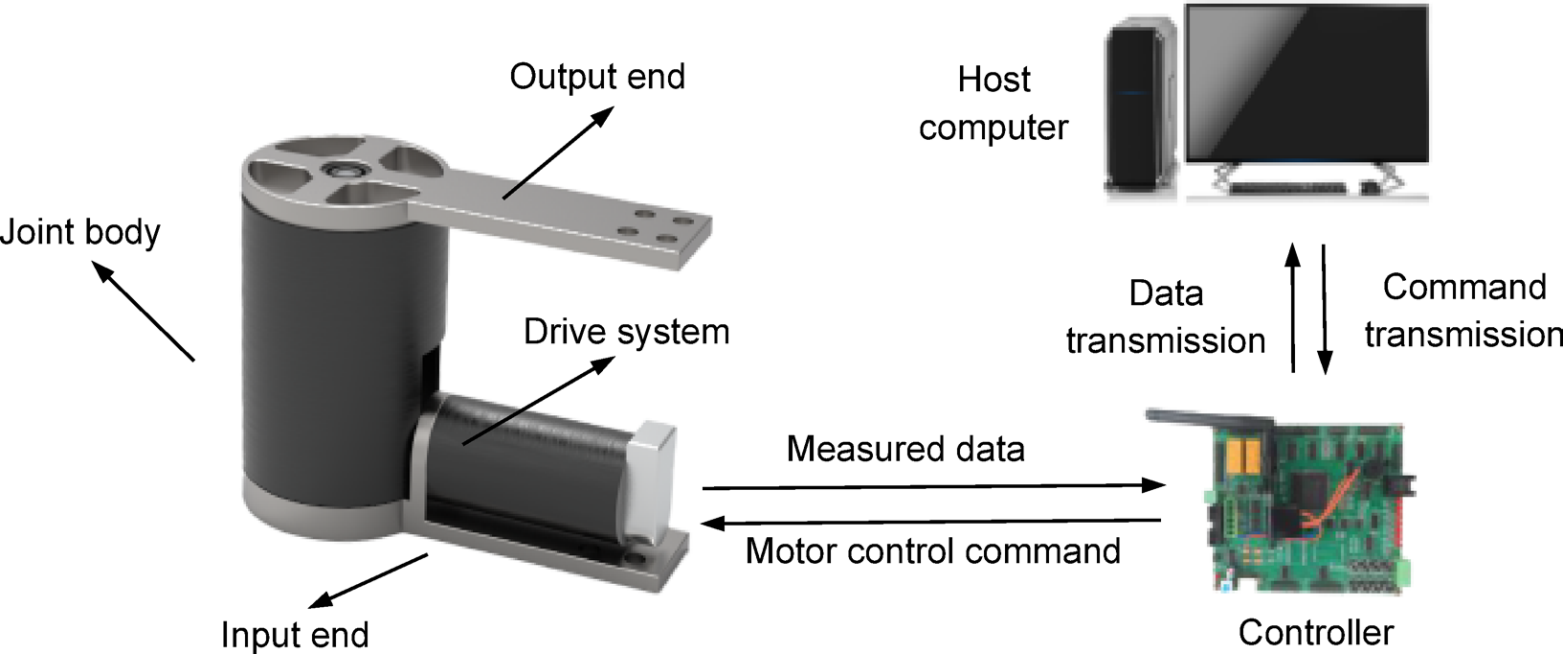
Schematic diagram of the working principle of the variable-stiffness joint.

Figure 3 illustrates the overall conceptual framework of the proposed TSDV variable stiffness joint. The system mainly consists of two parts, namely, the mechanical structure and the drive system, and possesses one rotational degree of freedom. A drive motor module is arranged at the input end to actively regulate the input torque, while the output end is connected to the load to generate a compliant response. An angular sensor is installed at the output end for real-time measurement of the joint output angle. The joint is driven by a cable-driven mechanism, and a force sensor is mounted on the driving cable for real-time measurement of the cable tension. In addition, the drive system employs a servo motor to accurately control the rotation of the main shaft, thereby ensuring the accuracy and repeatability of the input process. The entire system operates through the coordinated action of a controller and a host computer: the host computer issues commands and receives feedback data, while the controller performs signal processing and real-time motor command execution, thereby achieving variable stiffness regulation and closed-loop control of the joint.

**Fig. 4:**
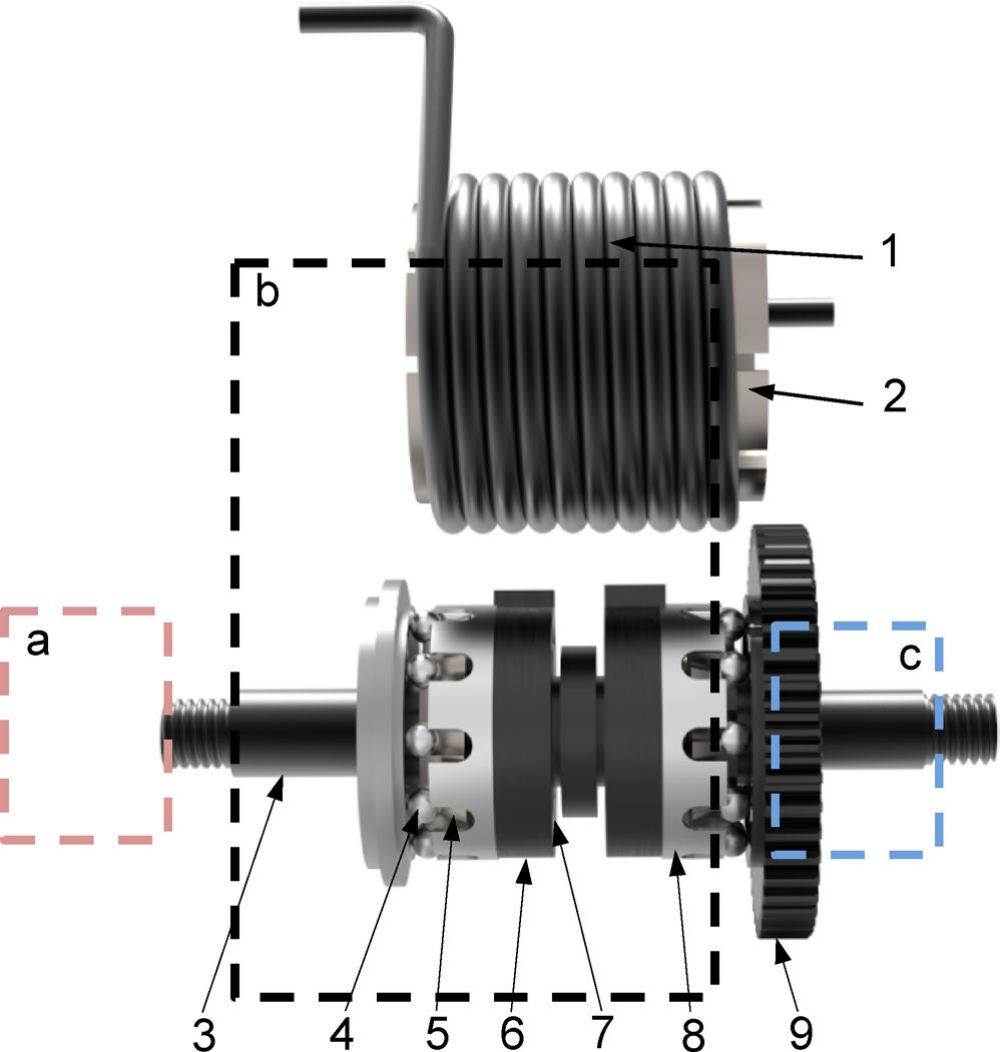
Structural schematic of the variable-stiffness joint. (**a**) input end; (**b**) joint body; (**c**) output end; (1) torsion spring; (2) slotted sleeve; (3) main shaft; (4) ball; (5) ball-guiding sleeve; (6) anti-rotation ring; (7) nut; (8) retainer; (9) gear.

A compressible slotted sleeve is arranged inside the torsion-spring joint. By means of force-transmission components such as the ball and ball-guiding sleeve, the contact interaction between the slotted sleeve and the inner side of the torsion spring is regulated, thereby enabling adjustment of the joint stiffness characteristics. The active torque of the joint is transmitted through a gear drive. As shown in Fig. 4, the mechanism mainly consists of a torsion spring, a slotted sleeve, a main shaft, balls, a ball-guiding sleeve, an anti-rotation ring, a nut, a retainer, and the gear.

The slotted sleeve 2 and the gear 9 on the right side enclose the internal transmission components within the torsion spring. The ball-guiding sleeves 5 arranged symmetrically on both sides are fitted to the main shaft 3 and can slide relative to it in the axial direction. Several grooves are uniformly distributed on each ball-guiding sleeve, and one ball 4 is mounted in each groove. The motion of the balls is jointly constrained by the slotted sleeve, the torsion spring, and the flange features of the gear. Because the threads at the two ends of the main shaft have opposite helix directions, the nuts 7 on both sides move towards each other when the main shaft rotates. Axial slots are formed in the slotted sleeve and engage with the protrusions on the anti-rotation ring 6, thereby preventing the nuts from rotating with the main shaft and ensuring that they move only in the axial direction. A combined disc-spring assembly is installed between each ball-guiding sleeve and the corresponding nut. The retainer 8 is arranged outside the balls to limit their range of motion and prevent them from falling out. This structural design not only improves the overall compactness of the joint, but also increases the output torque of the torsion spring through an appropriate transmission ratio. In addition, the drive motor is mounted on the input end of the joint, thereby reducing the rotational inertia of the system while ensuring that the joint axis remains collinear with the centerline of the torsion spring.

When the output link is subjected to an external torque and undergoes deflection, the inner diameter of the torsion spring decreases according to Eq. (4), which causes compressive contact between the spring and the slotted sleeve. Under the constraint of the ball-guiding sleeves, the balls move inwards, thereby increasing the torsional moment and producing a change in the stiffness of the joint system. This process corresponds to passive variable stiffness. When the deflection angle of the torsion spring is kept constant, the threaded shaft is driven to rotate by the motor, causing the two nut sliders to move in opposite directions. This motion further pushes the balls away from the center of the torsion spring, and the uniformly distributed load is transmitted to the torsion spring through the slotted sleeve, thereby increasing the resisting torque and modifying the stiffness of the joint system. This process corresponds to active variable stiffness.

In this design, the rolling motion of the balls on the inclined surfaces converts axial force into radial force. The left–right symmetric configuration improves the load-carrying capacity of the joint and reduces the energy-storage density of the elastic element. Moreover, by integrating the mechanical structure within the torsion spring, the joint size and mass are reduced, while the rotational axis of the joint remains unchanged.

### Theoretical analysis of the TSDV variable stiffness joint

**Fig. 5:**
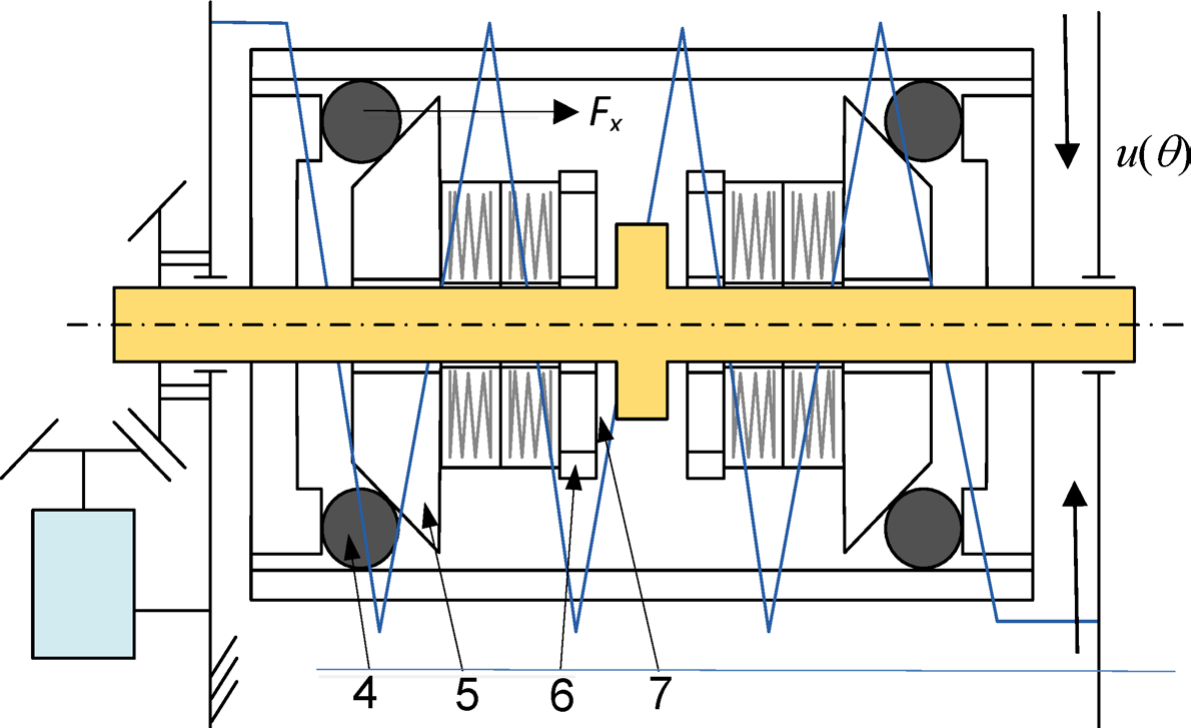
Schematic diagram of the mechanism, where the labels 4, 5, 6, and 7 are the same as those in Fig. 4.

When the input torque applied to the joint causes the torsion spring to undergo an angular displacement $\:\theta\:$, the inner diameter of the spring tends to contract, with the contraction amount denoted by $\:{\Delta\:}{R}_{i}\left(\theta\:\right)$. This contraction is constrained by the slotted sleeve. The radial compression of the sleeve, $\:u\left(\theta\:\right)$, generates an axial force component $\:{F}_{x}\:$through the rolling motion of the balls along the inclined surfaces, which in turn compresses the disc springs. The reaction force of the disc springs is then fed back to the sleeve and the torsion spring through the inclined surfaces and the balls, causing the equivalent stiffness of the joint to increase gradually with the deflection angle. The schematic illustration is shown in Fig. 5.

After the torsion spring exceeds its critical twisting angle, it continues to deform, and the reduction in the mean coil diameter produces a compression of the slotted sleeve. The compression amount $\:u\left(\theta\:\right)\:$can be expressed as $\:{\Delta\:}{R}_{i}\left(\theta\:\right)-{\delta\:}_{0}$, where $\:{k}_{c}\:$denotes the equivalent radial stiffness of the contact between the inner surface of the torsion spring and the sleeve.

The elastic potential energy stored in the compressed slotted sleeve can be written as:

6 $${W_{c\theta }}(\theta )=\frac{1}{2}{k_c}{u^2}(\theta )$$

Define the inner diameter variation function as:

7 $$f_{1} (\theta ) = \Delta R_{i} (\theta ) = \frac{{\theta D_{1} }}{{2(2\pi n_{1} + \theta )}}$$

Then, the compression amount $\:u\left(\theta\:\right)$can be expressed as $\:{f}_{1}\left(\theta\:\right)-{\delta\:}_{0}$.

Using the energy method, the additional torque generated by the compression of the slotted sleeve can be written as:

8 $${M_{c\theta }}(\theta )=\frac{{d{W_{c\theta }}}}{{d\theta }}={k_c}u(\theta ){f^{\prime}_1}(\theta )$$

The linear stiffness and torque of the torsion spring itself can be expressed as:

9 $${k_0}=\frac{{{E_s}{d^4}}}{{10.8{D_1}{n_1}}},{M_0}(\theta )={k_0}\theta$$

Assume that the number of balls is $\:N$, the slope angle of the ball guide sleeve is denoted by $\:\beta\:$, the coefficient of friction between the balls and the inclined surface is $\:{\mu\:}_{b}$, and the external force applied in the horizontal direction is $\:{F}_{d}$.

Then, the force component acting along the inclined surface on each ball can be expressed as:

10 $${F_p}={N_n}\sin \beta - {\mu _b}{N_b}\cos \beta$$

According to Eq. (10), the normal force $\:{N}_{b}\:$can be obtained as:

11 $${N_b}=\frac{{{F_d}}}{{N(\sin \beta - {\mu _b}\cos \beta )}}$$

The radial force acting on a single ball can be expressed as:

12 $${Q_b}={N_b}\cos \beta$$

By summing the radial force components of all the balls, the total radial force $\:{F}_{b0}\left(F\right)$ can be obtained as:

13 $${F_{b0}}(F)=N{Q_p}=\frac{{{F_d}\cos \beta }}{{\sin \beta - {\mu _b}\cos \beta }}=\lambda {F_d}$$

where $\:\lambda\:$ is a dimensionless constraint coefficient.

The radial force is converted into a constant radial compression $\:{u}_{F}$ through the radial stiffness $\:{k}_{c}$ of the slotted sleeve.

14 $${u_F}=\frac{{{F_{b0}}(F)}}{{{k_c}}}=\frac{{\lambda F}}{{{k_c}}}$$

The radial compression of the slotted sleeve is equivalently modeled as a linear spring, and the corresponding elastic potential energy can be expressed as:

15 $${W_c}(u)=\frac{1}{2}{k_c}{u^2}$$

Within the contact region between the torsion spring and the slotted sleeve, the torsion spring tends to generate a contraction $\:s\left(\theta\:\right)$ according to the geometric relationship, whereas the actual compression of the slotted sleeve is only $\:u$, the difference between the two is:

16 $$\Delta (\theta ,u)=s(\theta ) - u$$

This constraint induces additional circumferential tensile–compressive stress in the spring wire and leads to the storage of strain energy. By equivalently treating the active coils as a circular structure with radius $\:\stackrel{-}{R}$, the total circumferential length can be expressed as:

17 $$L_{c} = 2\pi \bar{R}n_{1}$$

The average circumferential strain caused by the reduction in radius can be written as:

18 $$\varepsilon =\frac{\Delta }{{\bar {R}}}$$

Let the cross-sectional area of the spring wire be $\:A=\frac{{\uppi\:}{d}^{4}}{4}$. Then, the additional strain energy caused by the constraint can be expressed as:

19 $${W_s}=\frac{1}{2}{E_s}A{\varepsilon ^2}{L_c}$$

Substituting Eqs. (17) and (18) into Eq. (19) and rearranging yields:

20 $$W_{s} = \frac{{\pi n_{1} E_{s} A}}{{\bar{R}}}\Delta ^{2} = \frac{1}{2}k_{s} \Delta ^{2}$$

The torsional potential energy of the torsion spring itself is expressed in a linear form as:

21 $${W_0}(\theta )=\frac{1}{2}{k_0}{\theta ^2}$$

The potential energy term of the radial support force $\:{Q}_{F}$ with respect to the generalized displacement $\:u$ is written as:

22 $${W_F}(u)={Q_F}u=\lambda {F_d}u$$

Therefore, the total potential energy function of the system within the contact region can be expressed as:

23 $$W(\theta ,u,F)={W_0}(\theta )+{W_c}(u)+\frac{1}{2}{k_s}{[s(\theta ) - u]^2}+{W_F}(u)$$

For given $\:\theta\:\:$and $\:F$, the actual displacement $\:u$is determined by the stationary condition of the potential energy:

24 $$\frac{{\partial W(\theta ,u,F)}}{{\partial u}}=0$$

Thus, it can be obtained that:

25 $$u((\theta ,F))=\frac{{{k_s}[{f_1}(\theta ) - {\delta _0}] - \lambda F}}{{{k_s}+{k_c}}}$$

The total rotational torque of the system is defined as the partial derivative of the total potential energy with respect to $\:\theta\:$:

26 $$M(\theta ,F)={\left. {\frac{{\partial {W_0}(\theta ,u,F)}}{{\partial \theta }}} \right|_{u=u(\theta ,F)}}$$

Thus, it follows that:

27 $$M(\theta ,F)=\frac{{\partial {W_0}}}{{\partial \theta }}+{k_s}[s(\theta ) - u(\theta ,F)]\frac{{ds(\theta )}}{{d\theta }}$$

Substituting Eq. (25) into Eq. (27) and rearranging yields the explicit form:

28 $$M(\theta ,F)={k_0}(\theta )+\frac{{{k_s}}}{{{k_s}+{k_c}}}[{k_c}s(\theta )+{Q_F}]{f^{\prime}_1}(\theta )$$

The final expression indicates that the horizontal thrust suppresses the contraction of the slotted sleeve by increasing the radial supporting force, thereby enhancing the resistance to contraction and making the growth of joint output torque with respect to the torsion angle more pronounced, exhibiting an adjustable nonlinear characteristic.

**Fig. 6:**
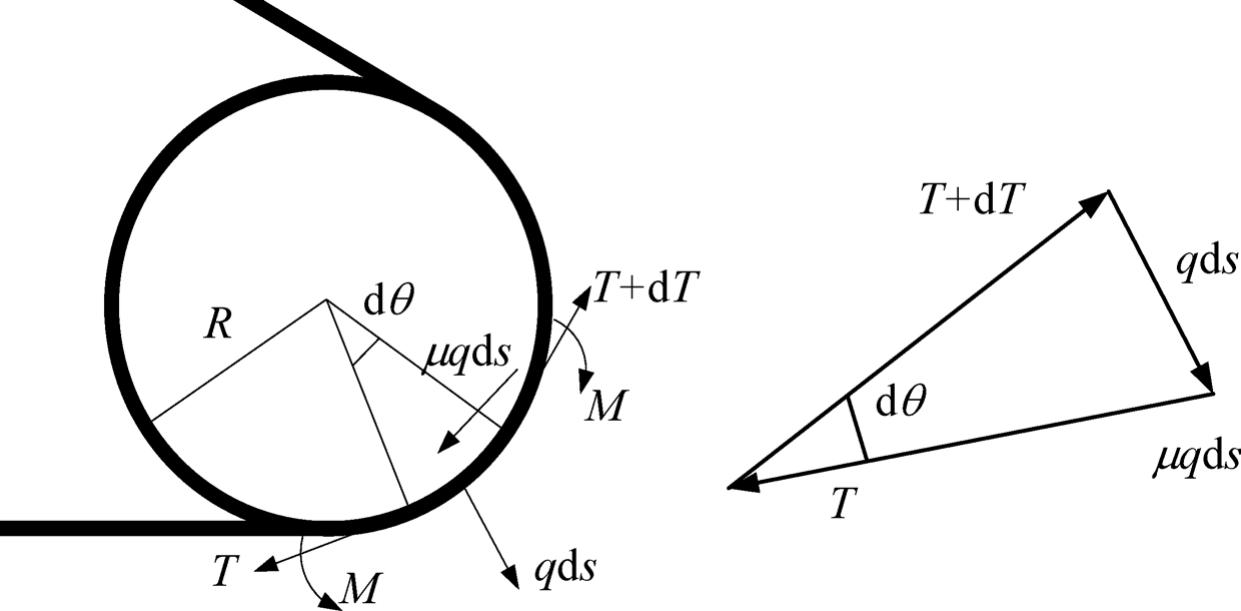
Force diagram of torsion spring.

When the spring is fitted over a rigid cylindrical shaft with the same inner diameter, an infinitesimal spring segment with length $\:ds$ is isolated as a free body, as shown in Fig. 6.

According to the equilibrium condition, it follows that:

29 $${\mathrm{d}}T=\mu q{\mathrm{d}}s=\mu T{\mathrm{d}}\theta$$

Integrating yields the following expression:

30 $$\int_{{{T_1}}}^{{{T_2}}} {\frac{{{\mathrm{d}}T}}{T}=} \int_{{{\theta _1}}}^{{{\theta _2}}} {\mu {\mathrm{d}}\theta }$$

Furthermore, it can be obtained that:

31 $$\left. {\ln T} \right|_{{{T_1}}}^{{{T_2}}}=\left. {\mu \theta } \right|_{{{\theta _1}}}^{{{\theta _2}}}$$

Thus, it follows that:

32 $${T_2}={T_1}{e^{\mu ({\theta _2} - {\theta _1})}}$$

Equation (32) shows that the tensile force variation of the spring wire wound around the cylindrical shaft conforms to the Euler formula. Under a driving torque applied in the helical winding direction, the spring mounted on the cylindrical shaft tends to rotate. However, friction prevents the spring coils from rotating immediately, and the spring is therefore tightened. With the increase in driving torque, the tensile force in the spring wire and the radial distributed pressure acting on it both increase, leading to a corresponding increase in the friction between the spring coils and the cylindrical shaft. As a result, the spring engages with the cylindrical shaft and rotates together with it. At this stage, the relative rotational degree of freedom of the joint is effectively constrained, and therefore this state can be equivalently regarded as a purely rigid joint.

## Results

Based on the stiffness model expression of the variable stiffness joint using a torsion spring, the relationships between joint output torque and deflection angle, as well as between joint stiffness and deflection angle, are analyzed. The component parameters are listed in Table 1.

**Table 1:** Structural parameters of the variable stiffness joint.

Parameter	Meaning	Value	Unit
*k*_*c*_	The equivalent radial stiffness	300	N/mm
*β*	Slope angle of the ball guide sleeve	60	^o^
*N*	Number of balls on one side	20	-
*D*_1_	Mean diameter of the torsion spring	38	mm
*n*_1_	Number of effective coils of the spring	10	-

**Fig. 7:**
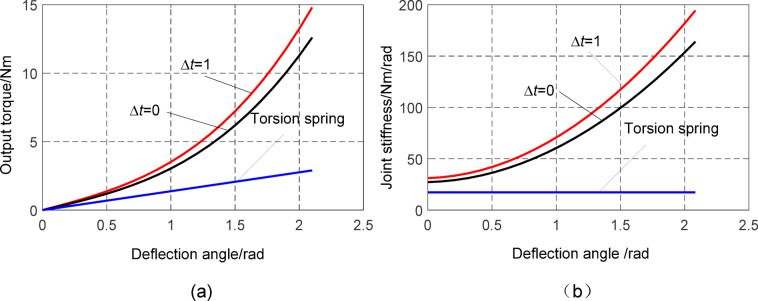
TSDV passive stiffness modulation mode. (**a**) Output torque curve, and (**b**) joint stiffness curve.

**Fig. 8:**
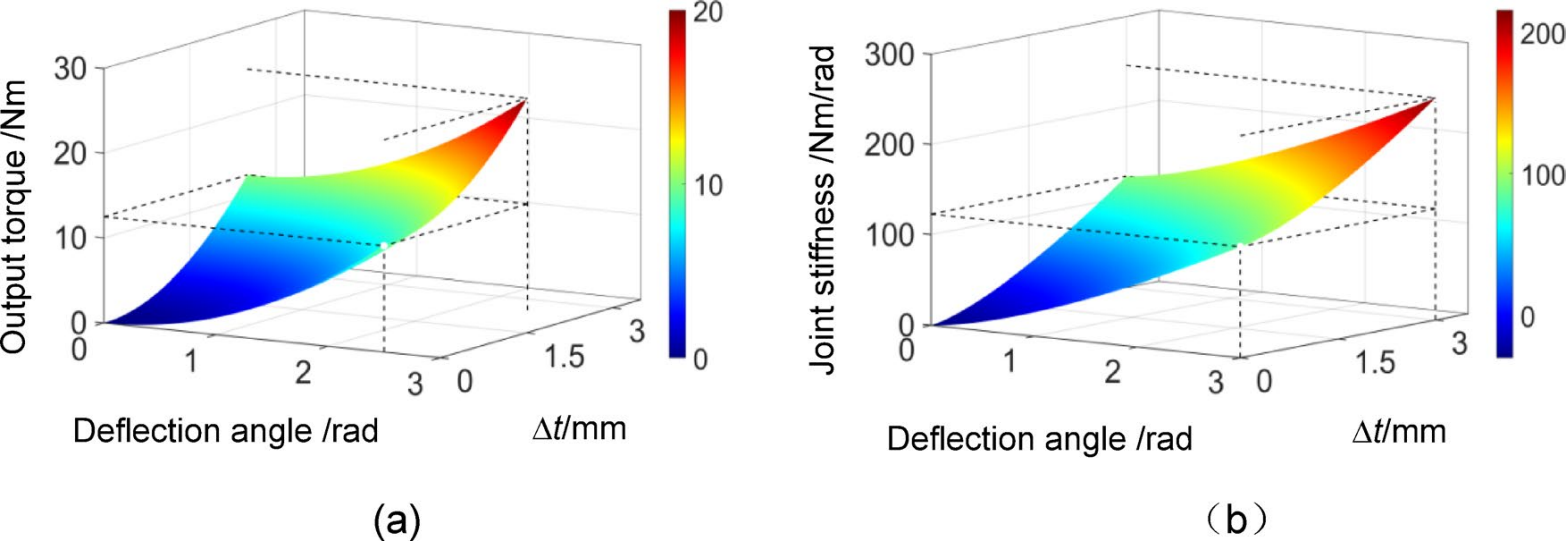
TSDV active stiffness modulation mode. (**a**) Output torque curve, and (**b**) joint stiffness curve.

According to the blue curve in Fig. 7, the torque of the torsion spring increases with the joint deflection angle, and the relationship between them is linear, with a constant stiffness of 16.1 Nm/rad. The black curve represents the output torque–deflection angle relationship at the initial working position, where the inner coil of the torsion spring is in a critical contact state with the slotted sleeve after the internal threaded shaft of the TSDV rotates by a certain angle. As shown in Fig. 7, after a deflection of 120°, the torque variation range increases from 0 to 2.7 Nm to 0–12.5 Nm, and the relationship between the joint output torque and deflection angle becomes nonlinear. As the deflection displacement increases, the stiffness rises from 16.1 Nm/rad to 163.5 Nm/rad. When the threaded shaft rotates such that $\:{\Delta\:}t=1$mm, the red curve in Fig. 7 shows that both the output torque and the joint stiffness of the TSDV increase further.

According to the calculated results shown in Fig. 8, At the same deflection angle of the variable stiffness joint, a greater output torque can be obtained with an increase in the axial displacement increment Δt caused by the main shaft rotation. Because the change in Δt requires real-time regulation by the driving motor, this process is characterized as active variable stiffness. The deflection angle–stiffness results in Fig. 8(b) indicate that the stiffness increases monotonically in both the passive and active stiffness adjustment modes. In addition, the coordination between the active and passive stiffness regulation modes allows the joint stiffness corresponding to different deflection angles to be adjusted, thereby improving the applicability of the proposed variable stiffness joint.

**Fig. 9:**
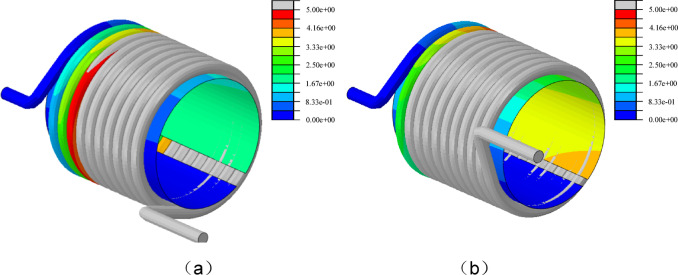
Finite element simulation results. Panels show the displacement contours of the slotted sleeve at deflection angles of (**a**) 60° and (**b**) 120°.

**Fig. 10:**
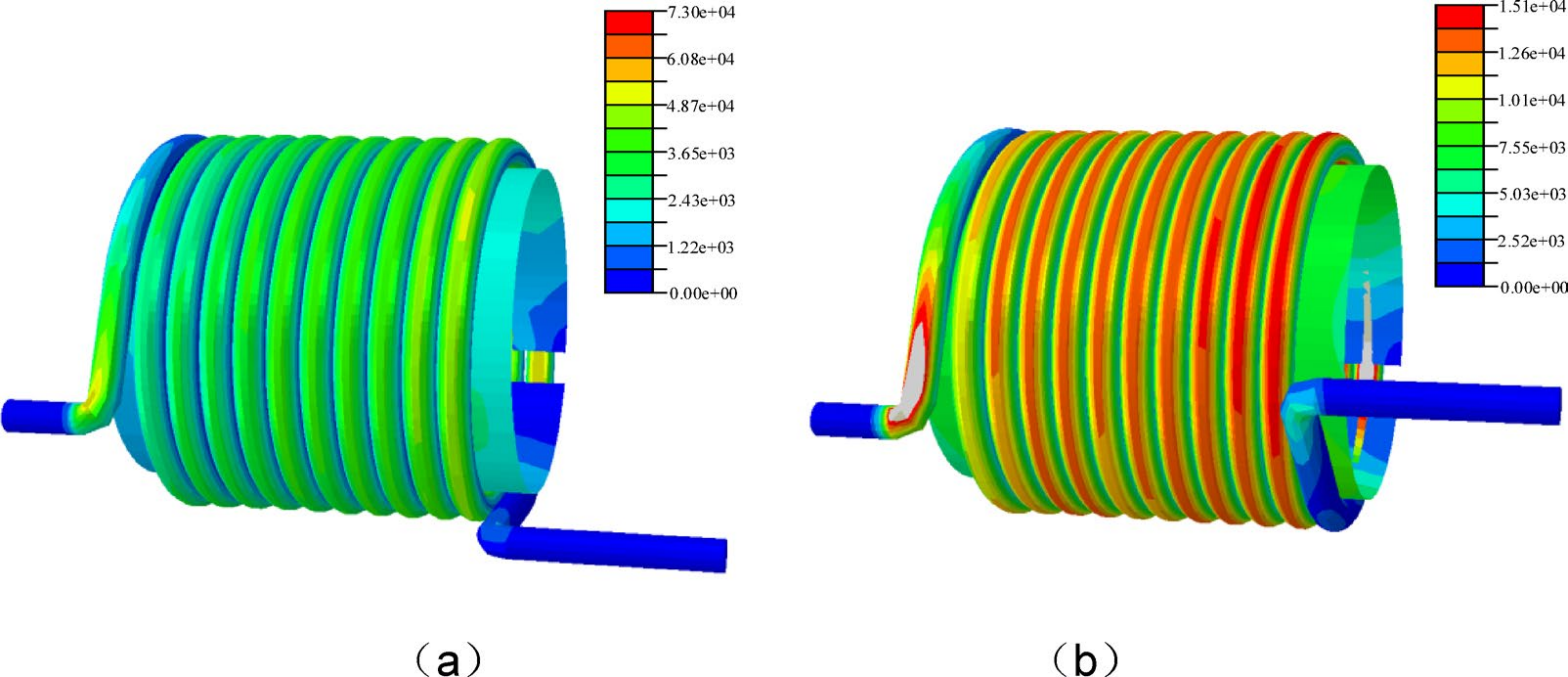
Finite element simulation results. Panels show the stress distributions of the torsion spring at deflection angles of (**a**) 60° and (**b**) 120°.

The correctness of the established mechanical model is verified using the finite element method. According to the previously proposed structural design, a virtual prototype model is constructed. First, the following assumptions are made: (1) the material is uniformly distributed within the object, meaning that the material properties are identical at all locations, and the material description is the same throughout the structure; (2) the contact stress between the torsion spring and the slotted sleeve is assumed to be uniformly distributed. The torsion spring was assigned an elastic modulus of 206 GPa and a Poisson’s ratio of 0.30. Surface-to-surface contact was defined between the inner surface of the torsion spring and the outer surface of the slotted sleeve. In the normal direction, the Hard Contact algorithm was adopted, while in the tangential direction, the friction coefficient was set to 0.15. In the simulation, one end of the torsion spring was fixed, and an incrementally increasing angular displacement load was applied to the other end, with a maximum imposed rotation angle of 120°. During meshing, local refinement was performed in the contact region, and the element type C3D8R, an eight-node linear brick reduced-integration solid element, was used.

**Fig. 11:**
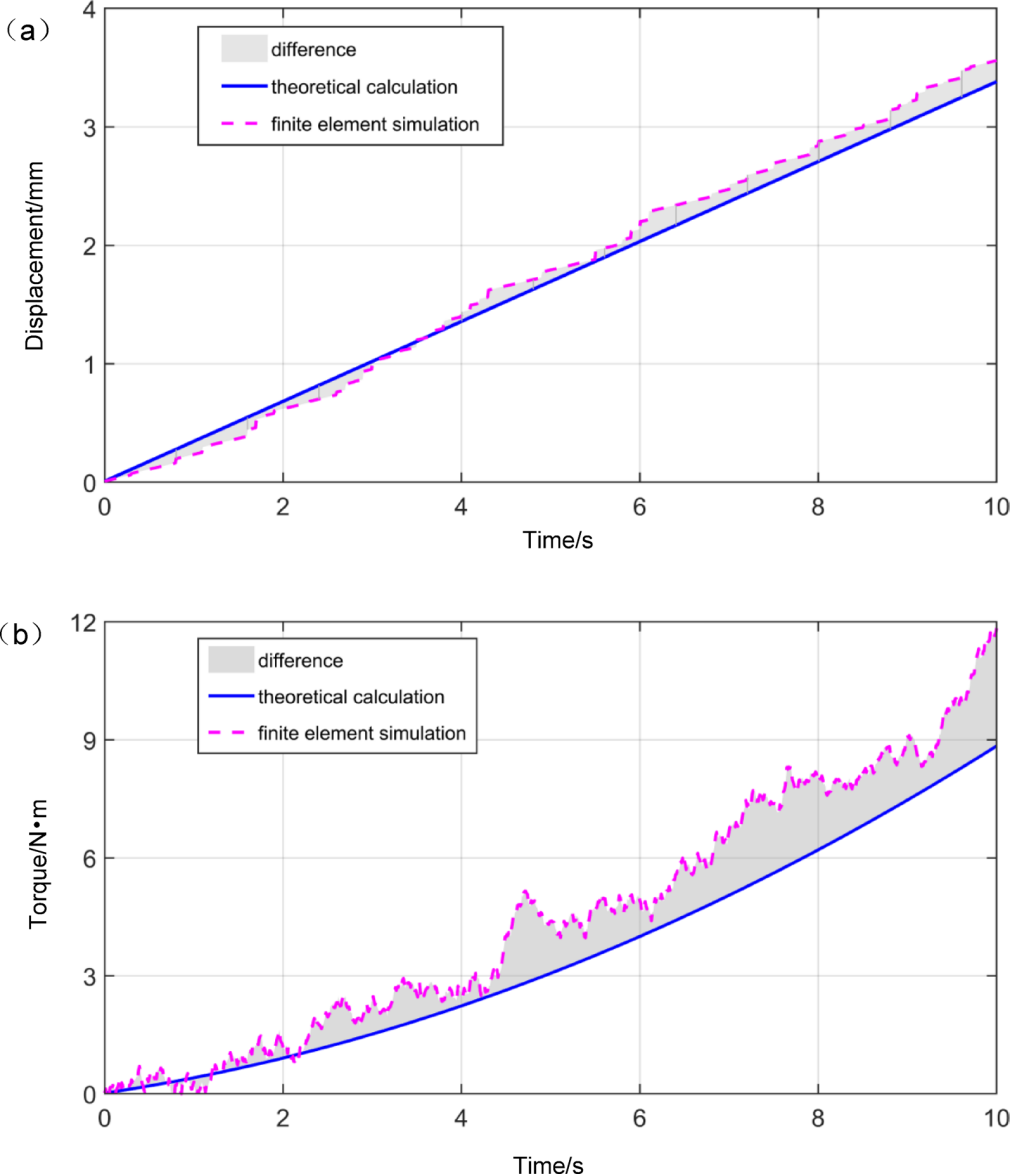
Comparison between theoretical calculation and finite element simulation results. Panels show (**a**) the displacement–time curves and (**b**) the torque–time curves.

Figures 9 and 10 show the displacement and torque variations of the torsion spring at deflection angles of 60° and 120°, respectively. A finite element node on the slotted sleeve is selected, and its displacement relative to the centerline is measured, as shown in Fig. 11(a). The results indicate that the maximum error between the theoretical value calculated by the model and the numerical value obtained from the simulation is 0.23 mm, while the overall variation trends are consistent. The main source of the error is that the slotted sleeve is modeled as an equivalent thin-walled structure.

A finite element node at the end of the torsion spring was selected to measure the torque variation curve, as shown in Fig. 11(b). The results indicate that the theoretical values calculated by the model are generally consistent with the simulation results in terms of the overall variation trend. When the torsion angle exceeds 60°, however, the discrepancy between the two increases, with a maximum error of 2.9 Nm, and the simulated torque is noticeably larger than the theoretical value. The main reason for this error is that the friction between the spring wires increases, causing the uniformly distributed load in the simulation to exceed the preset value.

## TSDV prototype testing experiment

**Fig. 12:**
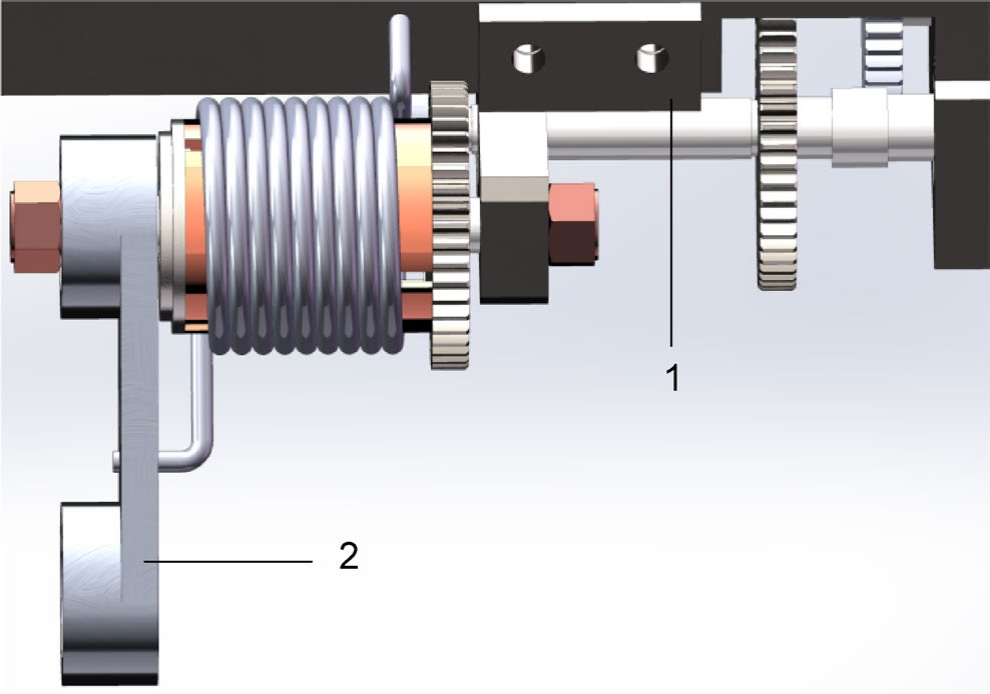
Three-dimensional assembly design of the TSDV. (1) is the joint input end, and (2) is the joint output end.

The overall assembly design of the TSDV is shown in Fig. 12, where component 1 represents the input end of the joint, on which the motor and reducer are mounted and which is connected to the fixed base. Component 2 denotes the output link of the joint, which is connected to the robotic arm and can achieve pose adjustment under external actuation. An angle sensor is also mounted on the main body of the output link to measure the deformation angle of the joint.

**Fig. 13:**
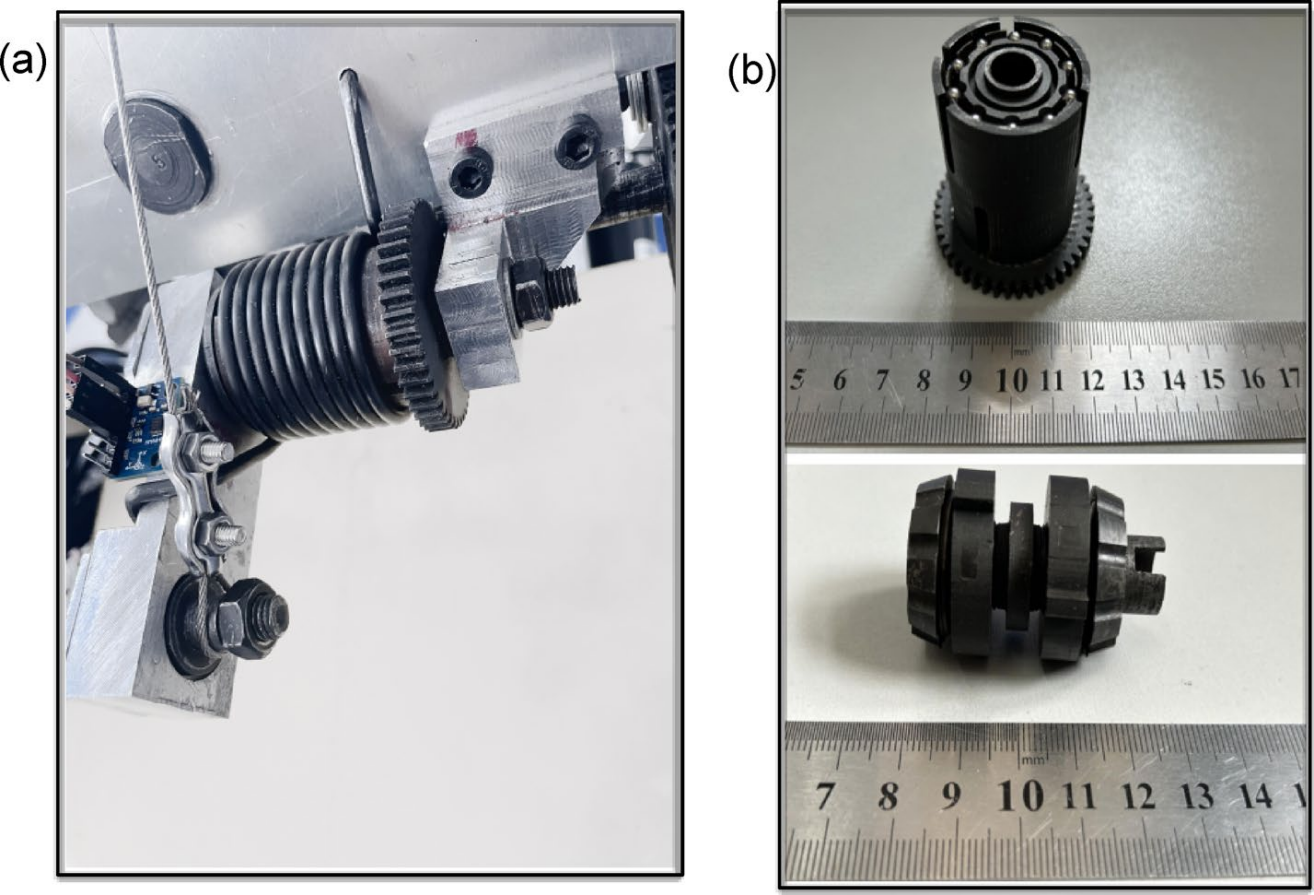
Physical prototype of the TSDV. Panels show (**a**) the overall prototype view and (**b**) the internal components view.

The TSDV joint prototype is shown in Fig. 13. Its overall assembly adopts a general and compact structural design to facilitate the assembly of the robot prototype and the subsequent replacement of vulnerable components. The main parameters of the TSDV prototype are listed in Table 2.

**Table 2:** Main design parameters of the TSDV.

Parameter	Value	Unit
Overall dimensions	Φ75 × 100	mm
Weight of a single joint	0.8	kg
Maximum compliant deformation angle	120	o
Minimum output stiffness	20	Nm/rad
Maximum output stiffness	≈+∞	Nm/rad

To decouple stiffness modulation from joint pose adjustment, the central threaded shaft of the TSDV was designed as a hollow tube that is mechanically separated from the internal rotating main shaft, as shown in Fig. 13b. The slotted sleeve was fabricated from 55CrSi spring steel because of its excellent fatigue resistance. It has a thickness of 2 mm and was formed by cold coiling. The groove of the ball guide sleeve was hard-anodized to increase surface hardness. Because this component is subjected to relatively large radial loads under low sliding speeds, it was paired with a quenched threaded shaft to enhance radial load-carrying capacity. The ball guide sleeve has a large-end diameter of 27 mm, a small-end diameter of 23 mm and a thickness of 5 mm. A threaded shaft with a pitch of 1 mm was adopted to improve the resolution of stiffness regulation. The elastic element was a cylindrical torsion spring made of carbon spring steel, with an outer diameter of 42 mm and a wire diameter of 3.5 mm, and it was installed with a prescribed preload angle.

**Fig. 14:**
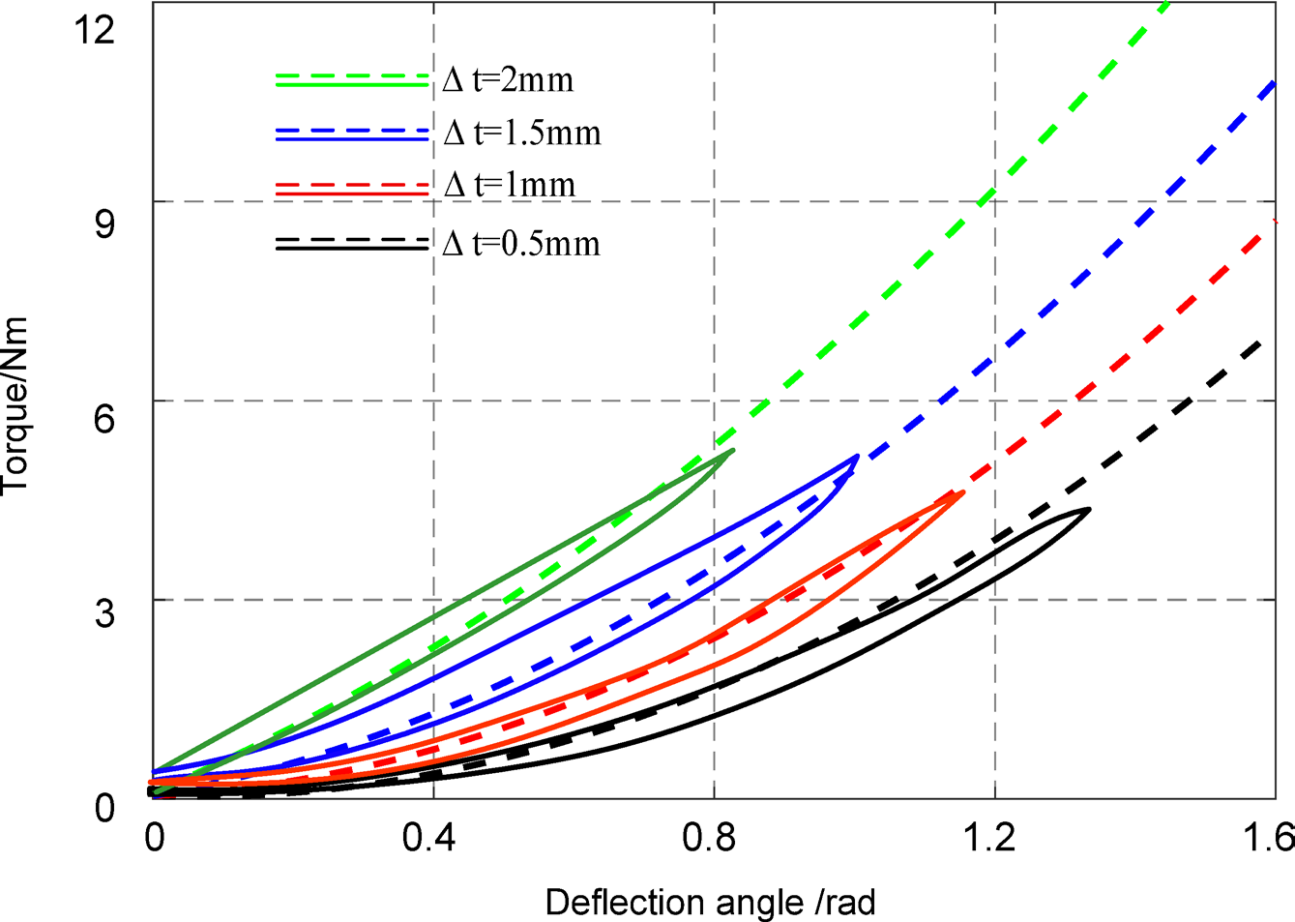
Output torque curve of TSDV.

During the passive stiffness modulation experiment of the TSDV, a cable was used to drive the output link to rotate slowly at a constant speed, while the stiffness regulation motor remained stationary. The internal slider was always kept at its initial position, and the relative displacement remained unchanged. A triaxial accelerometer mounted on the output link was used to measure the joint deformation angle of the TSDV, while a cable tension sensor was used to acquire the joint tensile force. Combined with the geometric relationship, the joint output torque was then calculated. The analysis and verification of the joint stiffness in this study are mainly based on the torque–angle relationship and its slope variation, on the basis of which the joint stiffness results are further obtained. In this way, the relationship curve between the joint deflection angle and the output torque of the TSDV in the passive stiffness modulation mode was obtained, as shown in Fig. 14. The tangent slope of each curve represents the joint stiffness. It can be seen from the curves that, when $\:{\Delta\:}t$ remains unchanged, the passive stiffness of the joint gradually increases, which is consistent with the design expectation. In addition, the curves of joint output torque versus angle were plotted for four cases, namely $\:{\Delta\:}t=0.5$mm, 1 mm, 1.5 mm, and 2 mm. The results show that the objective of active stiffness regulation can be achieved by actively adjusting the slider position. When the angle is 1.2 rad, the corresponding range of joint output torque increases from 0 to 3.8 Nm to 0–9.2 Nm. As $\:{\Delta\:}t$ increases, the slope of the torque–time curve also increases, indicating a higher rate of stiffness variation and thereby verifying the active variable stiffness characteristic of the TSDV. The dashed lines in the figure represent the theoretical calculation results, while the solid lines in different colors denote the loading and unloading experimental curves. The experimentally measured torque–deflection curves are in good agreement with the theoretical results, which verifies that the TSDV has high stiffness regulation accuracy. The fact that the solid line does not return along the original path but instead forms a closed loop is caused by the inconsistency in the torque–angle relationship during loading and unloading. The possible reasons are as follows: (1) friction in the contact pairs. The mechanism includes multiple contact pairs, such as balls, inclined planes, guide sleeves, and slotted sleeves, which results in different friction directions and contact force states during loading and unloading. (2) assembly clearance. Small assembly clearances exist in the mechanism, causing the internal components of the torsion spring to come into contact in a different sequence during loading and unloading.

**Fig. 15:**
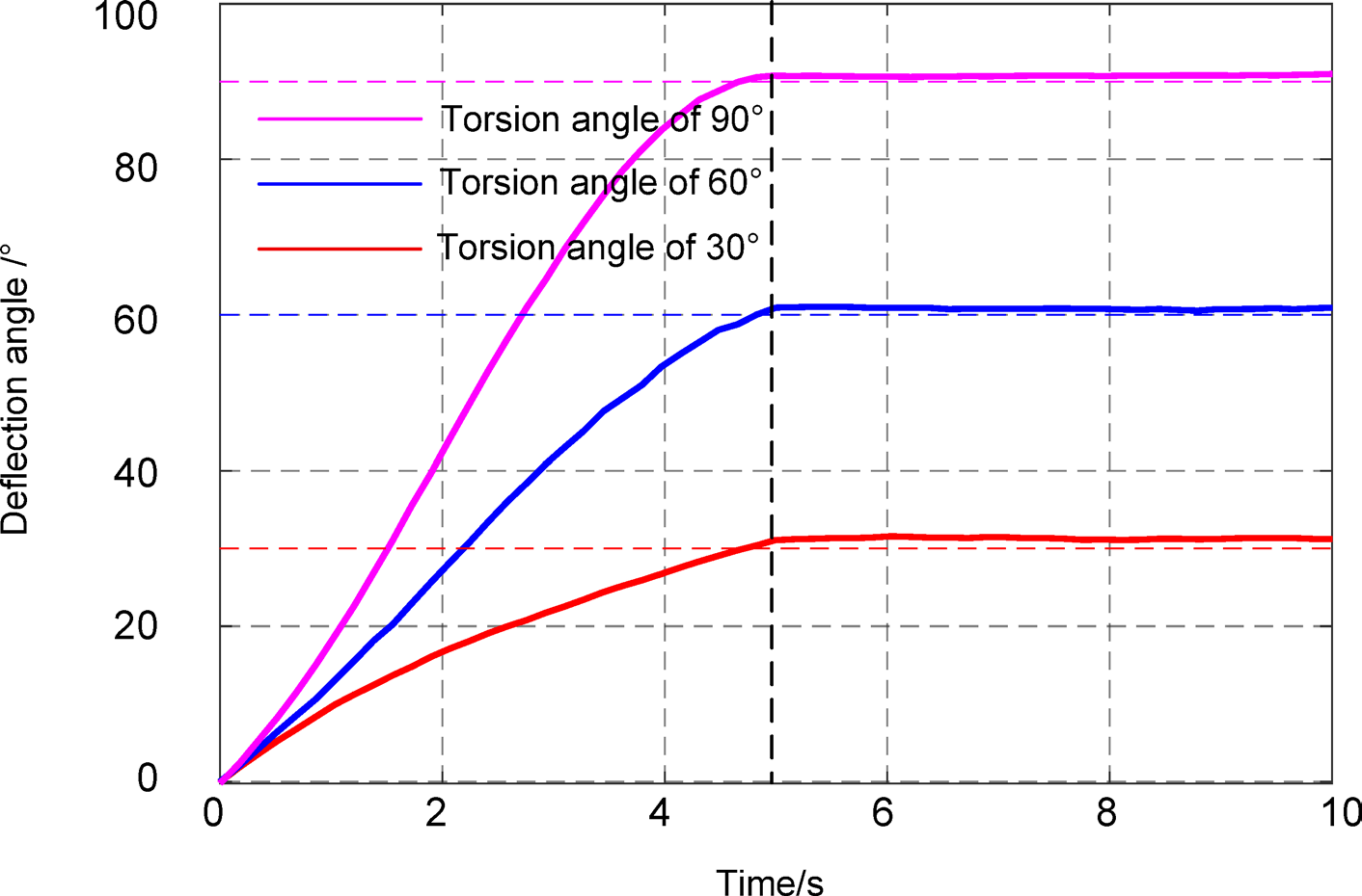
Experimental locking test of the TSDV.

To verify that the designed variable stiffness joint can increase its stiffness to infinity, the experiment was conducted at joint deflection angles of 30°, 60°, and 90°. In each case, the driving motor was used to adjust $\:{\Delta\:}t$to its maximum value while keeping the horizontal mechanism in a self-locking state. Under this condition, the slotted sleeve supports the inner coil of the torsion spring and restrains the variation of its mean diameter. The cable tension was then gradually increased until the threshold of the tension sensor was reached. As shown in Fig. 15, after 5 s, the joint angle no longer increased with the increase in tension, and a certain error existed between the link angle and the desired angle. When the joint angle was 30°, the maximum deviation was 1.4°. The reasons for this deviation include two aspects: (1) elastic deformation of the torsion-spring-based variable stiffness joint caused by the excessive length of the extended end of the torsion spring; and (2) assembly clearance resulting from machining errors of the mechanical parts. At this stage, the connection between the input link and the output link can be regarded as a rigid connection, thereby achieving the intended joint-locking function in the active rehabilitation mode. The above experimental results demonstrate that the TSDV can meet the requirements of different application conditions through active stiffness modulation.

## Conclusions

To overcome the limitations of conventional fixed-stiffness robotic joints, which hinder the simultaneous achievement of compliance and control performance, this study proposes a TSDV variable-stiffness joint based on the mean-diameter variation mechanism of a torsion spring and systematically evaluates its performance through theoretical modeling, numerical analysis, and prototype experiments. The results show that the mean-diameter variation of a cylindrical torsion spring during twisting can provide an effective physical basis for variable-stiffness realization. On this basis, a compact joint structure integrating a slotted sleeve and a ball–inclined-surface mechanism was developed, enabling both passive stiffness enhancement and active stiffness regulation. Furthermore, a nonlinear output torque model of the joint system was established using an energy-based method, revealing how radial supporting force suppresses the contraction of the inner coil of the torsion spring and thereby affects the nonlinear growth of output torque. Numerical simulations and finite element analysis demonstrate that the proposed theoretical model can accurately capture the nonlinear mechanical behavior of the joint. Prototype experiments further verify the variable stiffness performance of the proposed design: active adjustment of the slider position significantly increases both output torque and joint stiffness; at a deflection angle of 1.2 rad, the output torque range increases from 0 to 3.8 Nm to 0–9.2 Nm; and in the locking test, the joint achieves an approximately rigid connection. Overall, the proposed TSDV joint realizes combined passive and active stiffness modulation, achieves a wide stiffness regulation range while maintaining a compact structure, and provides a new solution with strong engineering potential for compliant robotic joint design.

## Data Availability

The data presented in this study are available upon request from the corresponding author.
